# Evaluating the effectiveness of a structured, simulator-assisted, peer-led training on cardiovascular physical examination in third-year medical students: a prospective, randomized, controlled trial

**DOI:** 10.3205/zma001504

**Published:** 2021-09-15

**Authors:** David M. Kronschnabl, Christoph Baerwald, Daisy E. Rotzoll

**Affiliations:** 1 Leipzig University, Faculty of Medicine, LernKlinik Leipzig, Skills and Simulation Centre, Leipzig, Germany; 2 University of Leipzig, Department of Internal Medicine, Division of Rheumatology, Leipzig, Germany

**Keywords:** peer-assisted learning, physical examination, simulation training, undergraduate medical education, clinical competence

## Abstract

**Background: **Previous research suggests that cardiac examination skills in undergraduate medical students frequently need improvement. There are different ways to enhance physical examination (PE) skills such as simulator-based training or peer-assisted learning (PAL).

**Aim: **The aim of this study was to evaluate the effectiveness of a structured, simulator-assisted, peer-led training on cardiovascular PE.

**Methods: **Participants were third-year medical students at Leipzig University Faculty of Medicine. Students were randomly assigned to an intervention group (IG) and a control group (CG). In addition to standard curricular training, IG received a peer-led, simulator-based training in cardiac PE. Participant performance in cardiac PE was assessed using a standardized checklist with a maximum of 25 points. Primary outcome was assessed via checklist point distribution.

**Results: **89 students were randomised to either CG (*n*=43) or IG (*n*=46) with 70 completing the study. Overall, IG students performed significantly better than CG students did (max. points: 25, *M*±*SD* in IG was 17±3, in CG 12±4, *p*<.0001). Simple mistakes such as not using the stethoscope correctly were more frequent in CG students. Prior experience did not lead to a significant difference in performance.

**Conclusions: **Structured, peer-led and simulator-assisted teaching sessions improve cardiac PE skills in this setting compared to control students that did not receive this training.

## Zusammenfassung

**Hintergrund:** Vorangegangene Untersuchungen deuten darauf hin, dass die kardiologische Untersuchungskompetenz von Medizinstudierenden häufig verbesserungswürdig ist. Es gibt verschiedene Möglichkeiten, die Fertigkeiten der körperlichen Untersuchung (KU) zu verbessern, wie z. B. simulatorgestütztes Training oder „Peer-Assisted Learning“ (PAL).

**Ziel: **Ziel dieser Studie war es, die Wirksamkeit eines strukturierten, simulatorgestützten und Peer-geleiteten Trainings bzgl. der KU des Herzkreislaufsystems zu evaluieren.

**Methoden:** Teilnehmende waren Medizinstudierende im ersten klinischen Jahr der Medizinischen Fakultät der Universität Leipzig. Die Studierenden wurden nach dem Zufallsprinzip einer Interventionsgruppe (IG) oder einer Kontrollgruppe (KG) zugeteilt. Zusätzlich zur curricularen Ausbildung erhielt die IG ein Peer-geleitetes, simulatorgestütztes Training in KU des Herzkreislaufsystems. Die Leistung der Teilnehmenden wurde unter Verwendung einer standardisierten Checkliste mit einem Höchstwert von 25 Punkten eingeschätzt. Der primäre Endpunkt war die mittels Checkliste vergebene Punktzahl.

**Ergebnisse:** 89 Studierende wurden in KG (*n*=43) oder IG (*n*=46) randomisiert, wobei 70 die Studie vollendeten. Insgesamt war die Leistung der Studierenden in der IG signifikant besser, als die der Studierenden in der KG (Höchstpunktzahl: 25, *M*±*SD* IG 17±3, KG 12±4, *p*<.0001). Einfache Fehler, wie die nicht korrekte Verwendung des Stethoskops traten bei Studierenden der KG häufiger auf. Vorerfahrung im medizinischen Bereich führte nicht zu einem signifikanten Unterschied in der Leistung.

**Schlussfolgerungen:** Strukturierte, Peer-geleitete, simulatorgestützte Lehrunterweisungen verbessern in diesem Setting kardiale KU-Fertigkeiten in der IG im Vergleich zur KG ohne ein solches Training.

## 1. Introduction

### 1.1. Background

The physical examination (PE) is a fundamental part of the diagnostic process and plays an important role even in modern day medicine [1]. According to previous studies skills in cardiac PE are low. Especially diagnostic accuracy in auscultation of cardiac murmurs and sounds is insufficient [2]. Cardiac examination skills do not improve significantly from third-year medical students to residents and faculty with the exception of cardiology fellows [3]. One study by Nielsen et al. [4] found a positive correlation of experience in auscultation with diagnostic specificity only, but not with diagnostic sensitivity. Cardiac auscultation diagnostic accuracy may actually decline with time after graduation [5].

#### 1.2. Simulators in medical education

Simulation technology is a valuable teaching tool in different medical fields [6], [7]. A life-sized cardiology patient simulator has been introduced and its value for teaching auscultation was tested with good results [8], [9]. Training using a cardiology patient simulator resulted in better cardiac examination skills compared with standard training when tested on standardized patients [10]. Successful transfer from simulation to real patients has been shown for cardiac auscultation [11], [12]. However, some studies suggest that simply adding a simulator to PE teaching is not sufficient to improve auscultation performance of undergraduate medical students [13].

#### 1.3. Peer-assisted learning (PAL) in medical education

In order to face new challenges in teaching clinical skills due to limited financial and human resources, new teaching approaches are needed [14]. One possible answer to some of these challenges is PAL [15]. Using PAL in medical education can save money and can be as effective as faculty-led training for advanced cardiac resuscitation [16], musculoskeletal system examination [17], technical procedures [18] and basic PE [19]. 

While PAL is well established in medical education, there is still need for further investigation under which circumstances it is effective [20].

To our knowledge there have been no randomized, controlled trials investigating the effectiveness of cardiac PE teaching for undergraduate medical students using near-peer tutors as teachers and a simulator as aid. 

#### 1.4. Aim of the study

The aim of this study was to evaluate the educational effectiveness of a structured, simulator-assisted, peer-led training on cardiovascular PE in third-year undergraduate medical students. Our main hypothesis was that this intervention would improve cardiac PE skills in the participants. We also investigated the influence of possible confounding variables, i.e. gender, age, prior experience, having the faculty-led PE skills course on a cardiology ward, having four or more of the five teaching sessions with the same faculty member, preparation for the skills assessment, time between the skills training and the skills assessment, time between the faculty-led PE skills course and the skills assessment.

## 2. Materials and methods

### 2.1. Study design 

**Figure 1:**
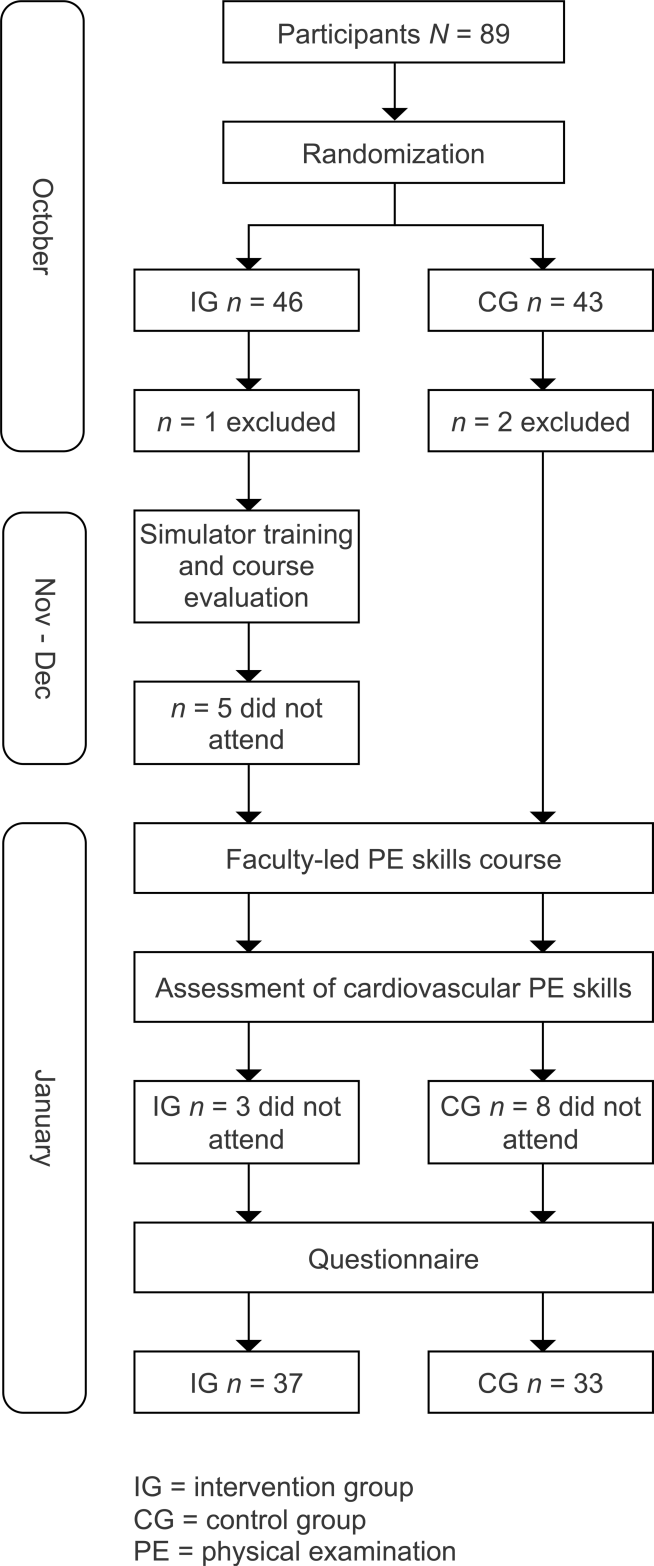
Study design

The study was a randomized, controlled trial. Figure 1 (Fig. 1) shows our study regimen und time schedule (see figure 1 (Fig. 1)). All participants were volunteer third-year undergraduate medical students who received an ID-number and were randomized (random numbers in Excel 2011 Version 14.1.0 for Mac) to either the intervention group (IG) or control group (CG). All IG students attended one of the simulator-based, peer-led training sessions on cardiovascular PE at the University of Leipzig Faculty of Medicine, Skills and Simulation Centre. The sessions of the simulator training were held in November and December and students were asked to evaluate the course afterwards. CG students did not receive this training before completion of the study. This training was the first teaching of practical physical examination skills for these students. In January all participants took part in the faculty-led course on history-taking and PE. This curricular course consisted of lectures and bedside-teaching sessions in different medical specialties. The bedside teaching on PE skills in internal medicine was divided in five ninety-minute sessions, for which students were assigned to the medical wards in study groups of n=4-6 students. In these sessions, cardiovascular PE was included among other topics such as history taking and examination of the abdomen, lungs, musculoskeletal system, thyroid gland and lymph nodes. There was no standardized script or schedule used by all faculty members on the wards. Therefore, contents of the course might have varied or had a different focus. Simulators were not used in this course. After the faculty-led PE skills course, the participants’ PE performance was assessed. Both groups were compared to assess the impact of the intervention. It should be noted that the results of the assessment had no influence on students’ grading of the curricular course. In order to detect other influences on PE performance, all participants completed a questionnaire (see attachment 1 ) asking for gender, age and prior experience in cardiovascular PE (e.g. prior training in healthcare professions, nursing placement or civil service on a cardiology ward, other practical experience in cardiovascular PE). Additionally, students provided information on the faculty-led PE skills course (which medical ward, how many of the five teaching sessions were held by the same faculty member, time spent on preparation and follow-up during the course) and on whether they prepared for the skills assessment. The questionnaire was anonymous and linked to the corresponding checklist using ID-numbers. 

#### 2.2. Simulator training with the Cardiology Patient Simulator (CPS)

The simulator training took place in a peer-led format, in groups of no more than six participating students (*n*=2-6, mean group size *M*=5,1) and lasted 75 minutes. In this course, the cardiology patient simulator “K” [21] was used. This CPS is able to simulate jugular venous waves, arterial pulses of carotid, brachial, radial and femoral arteries, cardiac impulses, respiratory sounds, abdominal respiratory movements as well as heart sounds and murmurs that all synchronize with each other. All heart sounds and murmurs are recordings from real patients. Only physiological cardiac simulations were used in the training. The focus of this training was on the technique and the systematic approach of cardiovascular PE with inspection, palpation, percussion and auscultation. Functions of the stethoscope and basic principles of cardiac auscultation were also explained. Inspection was discussed using photographic material depicting pathological findings. The students practiced taking peripheral pulses on each other. Precordial palpation and auscultation of physiological heart sound were practiced on the CPS. Learning objectives of the course are shown in table 1 (Tab. 1). The same peer student tutor taught all groups. The peer student tutor was a 4^th^-year medical student who had been trained in heart auscultation, cardiovascular PE and medical didactics. He had been tutoring heart auscultation and cardiovascular PE for over a year at the faculty’s Skills and Simulation Centre. 

#### 2.3. Skills assessment 

**Figure 2:**
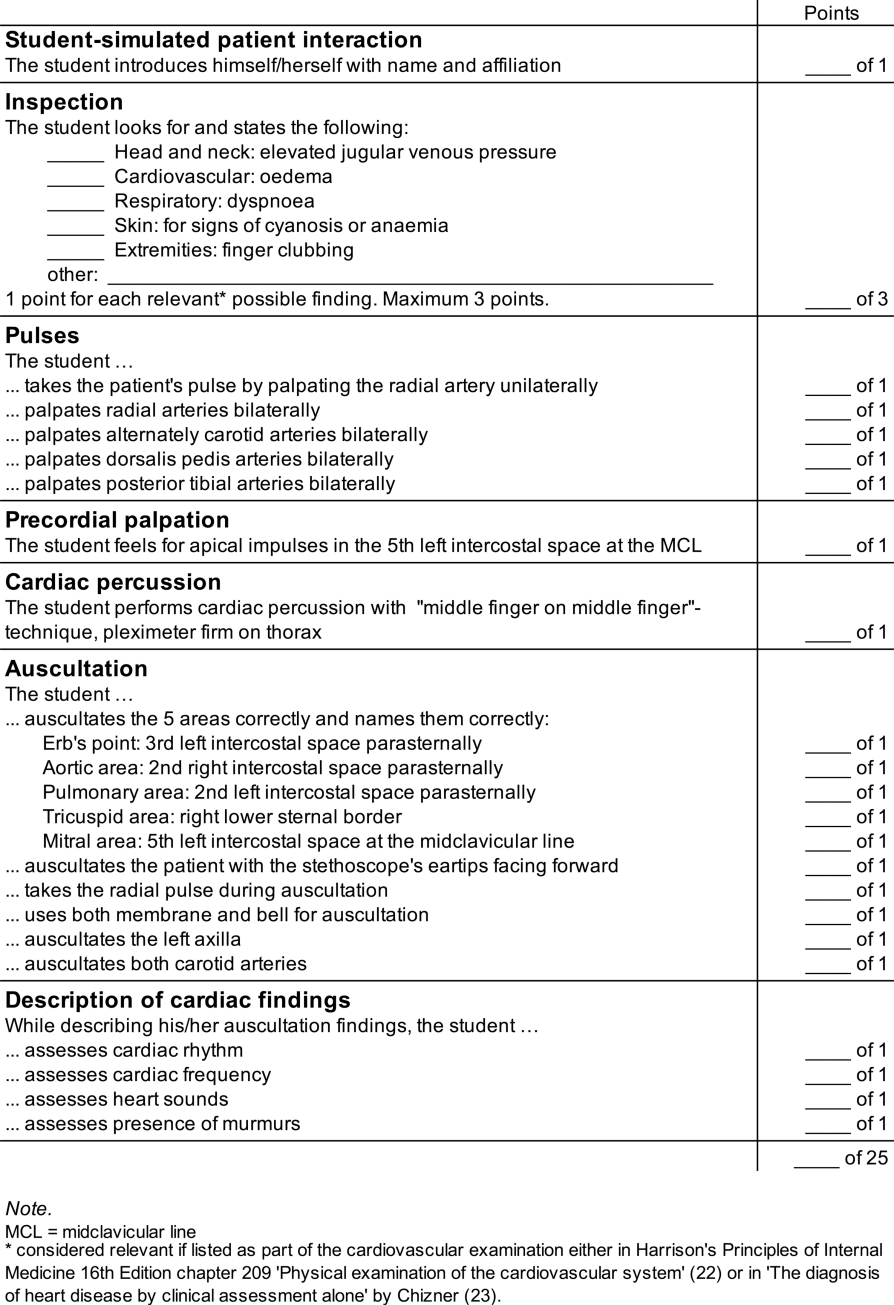
Cardiovascular physical examination, standardized assessment checklist

We assessed the participants’ performance using a standardized checklist (see figure 2 (Fig. 2)) [22], [23]. This checklist has been adapted from a cardiac findings checklist by Hatala et al. [24] to match the participants’ clinical knowledge. Our participants had received training in basic skills for PE, however, they had not yet received lectures or bedside teaching on cardiovascular diseases. Therefore, we assessed their PE technique only. 

In order to get an objective evaluation, the examiner was blind to the group assignment. The same observer (D.K.) assessed all participants.

For the skills assessment, all participants were asked to perform a PE of the cardiovascular system on a healthy male student while commenting on what they were examining and why they were doing the manoeuvre. The time limit was five minutes. If participants wanted to take a patient history or perform examinations not on the checklist, they were told to skip this part and carry on with the examination.

#### 2.4. Participants

The participants were third-year undergraduate medical students at the Leipzig University Faculty of Medicine (Leipzig, Germany), who had just completed two years of preclinical studies and had neither done any clinical electives, nor received curricular bedside-teaching in PE, nor been admitted to training in the faculty’s Skills and Simulation Centre on the subject matter. Students in training to be tutors at the Skills and Simulation Centre were excluded. With no previous exposure to PE skills and similar levels of previous experience in both groups, the same baseline level of competence in cardiac PE was expected. We obtained written informed consent for this study from all participants. 

#### 2.5. Data analysis

For statistical analysis, we used SPSS^®^ Version 22. For normal distribution testing Shapiro-Wilk test was used. The groups were compared with Mann-Whitney U-test for non-normally distributed data and Student’s *t*-test (independent samples) for normally distributed data. We performed Bonferroni’s correction for comparing group performance in the individual checklist categories. Fisher’s exact test was used for nominal data. Influences on the overall performance were examined with Student’s t-test and Spearman’s rank correlation coefficient (r_s_). Additionally, a multiple linear regression was conducted with total points as the dependent variable and possible influences as independent variables (enter method). Only predictors which we had formulated a hypothesis for, and which had no missing values, were included in the regression model. Variables that did not improve the adjusted *R^2^* were excluded. In the first step of the hierarchical regression, only the remaining confounders were entered as independent variables. In the second step, group assignment was added to the first model to calculate the *R^2^* change. 

A two-sided *p* value of <.05 was considered statistically significant. Continuous results are presented as mean (*M*)±standard deviation (*SD*).

## 3. Results

### 3.1. Descriptive data 

Out of *n*=89 students *n*=70 participants completed the study according to the protocol, *n*=33 in CG (23% dropouts) and* n*=37 in IG (20% dropouts) (see table 1 (Tab. 1)). The majority of drop outs were due to students not taking part in the assessment (IG *n*=3, CG *n*=8). Furthermore, in the IG *n*=5 additional students neither attended the simulator training nor the assessment. Beyond that, *n*=3 participants had started to work as tutors at the Skills and Simulation Centre (IG *n*=1, CG *n*=2) and were therefore excluded. We discovered no significant differences between the groups with respect to gender, age and prior experience. The majority of students in both groups spent less than 30 minutes on preparation and follow-up during the faculty-led PE skills course as well as on preparation for the skills assessment (see table 2 (Tab. 2)). 

#### 3.2. Students’ evaluation of the simulation training

IG students found the peer student tutor to be very competent (*M*=1.13±*SD*=0.34 on the 6-point Likert scale, with 1 meaning “I agree completely” and 6 meaning “I disagree completely”). They liked the structure of the course (1.16±0.37) and that the tutor was a student (1.18±0.46). The students reported to have benefitted from the peer student tutor’s feedback (1.53±0.73) and to have gained more security in cardiovascular PE (2.00±0.62). Almost all students found the training to be appropriate for third-year medical students, whereas only one found it slightly too easy. The theoretical aspects in the course were perceived to be very relevant for the practical exercises (1.45±0.65). In contrast, students did not agree to statements that they had already sufficiently observed (4.63±1.28), been shown and explained (4.47±1.13) or done themselves (5.47 ± 0.89) cardiovascular PE in their previous medical education. The students rated the course with the German school grade 1.21±0.43 (scale of 1 to 6 with 1 meaning “very good” and 6 meaning “insufficient”). The full report on the qualitative assessment can be seen online [25].

#### 3.3. Skills assessment and group comparison

**Figure 3:**
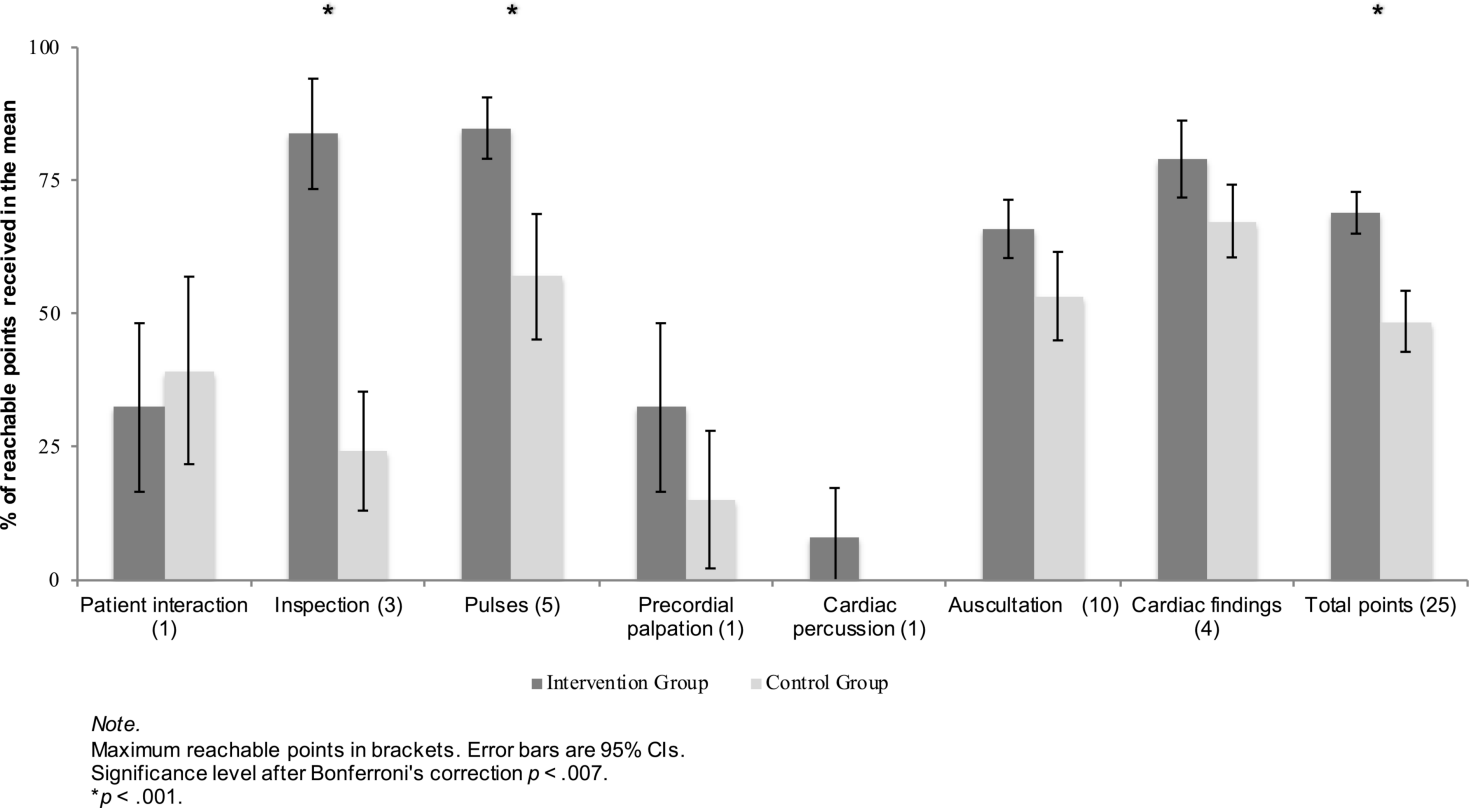
Group comparison in all checklist categories

IG students performed significantly better than CG students did (IG *M*=17.03±*SD*=3.01, CG 11.82±4.04, *p*<.0001). They also received more points for inspection and pulses. For most of the other checklist categories we saw a positive trend, but no statistically significant difference after Bonferroni’s correction (see figure 3 (Fig. 3)). 

Visual inspection of the patient was performed more frequently in the IG compared to the CG (IG 92%, CG 42%, *p*<.001). IG students looked for more possible findings (IG 3.35±1.57, CG 0.73±0.94, *p*<.001). They also palpated the four arteries (radial, dorsalis pedis, posterior tibial and carotid arteries) more frequently bilaterally (IG 3.42±0.86, CG 1.94±1.52, *p*<.001). IG as well as CG students did not use both membrane and bell of the stethoscope on a regular basis (24% vs. 12%), take pulses simultaneously (35% vs. 27%) or auscultate the left axilla (43% vs. 33%). 78% of IG students and 52% of the control group auscultated the carotid arteries. 27% of CG students used the stethoscope with the ear pits facing the back of the head, whereas only one student of the intervention group (=3%) made this mistake (*p*<.005).

#### 3.4. Influences on students’ performance 

We tested the possible confounders independently with Students’ *t*-test and could not detect any positive influence of prior experience, having the faculty-led PE skills course on a cardiology ward and having four or more of the five teaching sessions with the same faculty member (see table 3 (Tab. 3)). The influence of students’ preparation for the skills assessment could not be assessed adequately, because only two students had prepared for more than 30 minutes. A longer time between the simulator training and the skills assessment did not significantly correlate with lower mean results (*r_s_*=-.08; *p*=.65), whereas time between the faculty-led PE skills course and the skills assessment did (*r_s_*=-.26; *p*<.03). 

To correct for correlations between the confounding variables, a multiple linear regression was conducted as described above.

The histogram and scatterplot of standardized residuals showed approximately normally distributed errors and revealed no violations of the general assumptions of regression analysis. In addition, standard residuals showed no significant outliers (min=-2.22, max=2.47). Multicollinearity was no concern (tolerance >.9 for all variables; variance inflation factor ≤1.1 for all variables). Table 3 (Tab. 3) shows the final regression model and a significant *R^2^* change of .23 (*p*<.0001) for adding group affiliation to the first model. With regard to the possible confounding variables, the regression model had the same results as the individual analysis. Again, time in days between the faculty-led PE skills course and the skills assessment correlated with lower scores (β=-.19; *p*=.048). 

## 4. Discussion

Even today, cardiac physical examination skills are still essential for clinical decision making. Our data confirmed our basic hypothesis that these skills can be improved by addition of a structured, simulator-assisted, near-peer-led training to standard training. These results support the findings of Kern et al. [10], who have shown that simulator-based training can enhance cardiac PE skills in medical students when tested on healthy adults. In our study, this effect could be observed even 32-67 days after the intervention, suggesting a lasting positive effect. The simulator training featured PAL, simulator-assisted teaching and general principles of teaching like direct observation and clear learning objectives. Supervised hands-on practice opportunities with direct observation and feedback are crucial for developing PE skills, especially in the context of simulator-based teaching [6], [26]. The structure with clear learning objectives ensured that even the most basic aspects of cardiovascular PE, such as how to handle the stethoscope, were included in all teaching sessions. On the other hand, some teaching physicians might have taken this knowledge for granted. As a result, simple mistakes were less frequent in the intervention group. Additionally, IG students might have benefitted more from the curricular PE skills course than their peers, because they were feeling more comfortable [27], or the simulator course led to a better basis for the curricular course. 

We only tested cardiac PE technique because our students were still in the first clinical year. At a later stage of their training students should be tested for both PE technique and diagnostic accuracy because correct PE technique does not automatically lead to a high diagnostic accuracy [28]. 

The investigated possible confounders appear to have little impact on students’ performance in comparison to our intervention. This emphasizes the role of structured teaching in comparison to those confounders. It seems that prior experience without structured teaching is not a good predictor of cardiac PE skills. 

Students who had the curricular PE skills course on a cardiology ward scored lower points than students who had the course on a non-cardiology internal medicine ward (β=-.22, *p*=.03). The result does not prove lower-quality teaching on our cardiology wards, but rather suggests a different emphasis in teaching due to a lack of standardization. Furthermore, one should keep in mind that the curricular PE course in internal medicine consists of more than cardiac PE. 

Moreover, this study is in line with data on lacking PE skills in medical students [29], [30]. In a way, the unsatisfactory overall performance is understandable, because the students had only received one week of training, but Haring et al. [31] found that even after curricular training and a clerkship in internal medicine students often performed an incomplete physical examination. Omitting a relevant part of the physical examination has been linked to a wide variety of adverse effects [32]. Teaching basic PE skills should therefore remain a main concern in medical education. 

Regarding students’ evaluation of the simulator course, the results were not surprising. The positive feedback on the additional training supports previous findings that PAL is well received by students [33], [34]. Considering that the simulator training was held even before the curricular PE skills course, we also expected the students to report a lack of training and practical experience in PE so far. 

The main limitation of this study is the difference in total teaching time between the two groups. Since IG students received 75 minutes of additional training, our study cannot show that the intervention is superior to standard training. Nonetheless, the addition of peer-led, simulator-based training was successful in enhancing students cardiac PE performance. Furthermore, D.K., who assessed the participants’ performance, was a medical student who was trained in cardiac PE as a peer student tutor, and not a cardiologist. However, students have been used successfully as examiners in objective structured clinical examinations [35]. The transferability of our results is also limited by the small number of observations, the sample from only one medical school and one curriculum and the fact that the simulator training sessions were held by only one tutor. 

Suggestions for further studies include a three-armed study to compare this intervention with additional standard teaching and additional training with another modern teaching modality (e.g. e-learning-intervention) for a one-year cohort of n=320 students and with long-term follow-up. 

## 5. Conclusion

Skills of third-year undergraduate medical students in cardiovascular physical examination require improvement. The addition of structured, peer-led and simulator-assisted teaching sessions are an effective way to improve these skills. 

## Data

Data for this article are available from the Dryad Digital Repository: https://doi.org/10.5061/dryad.r7sqv9s8w [25]

## Acknowledgements

We thank all participating students. 

## Competing interests

The authors declare that they have no competing interests. 

## Supplementary Material

Student questionnaire

## Figures and Tables

**Table 1:**
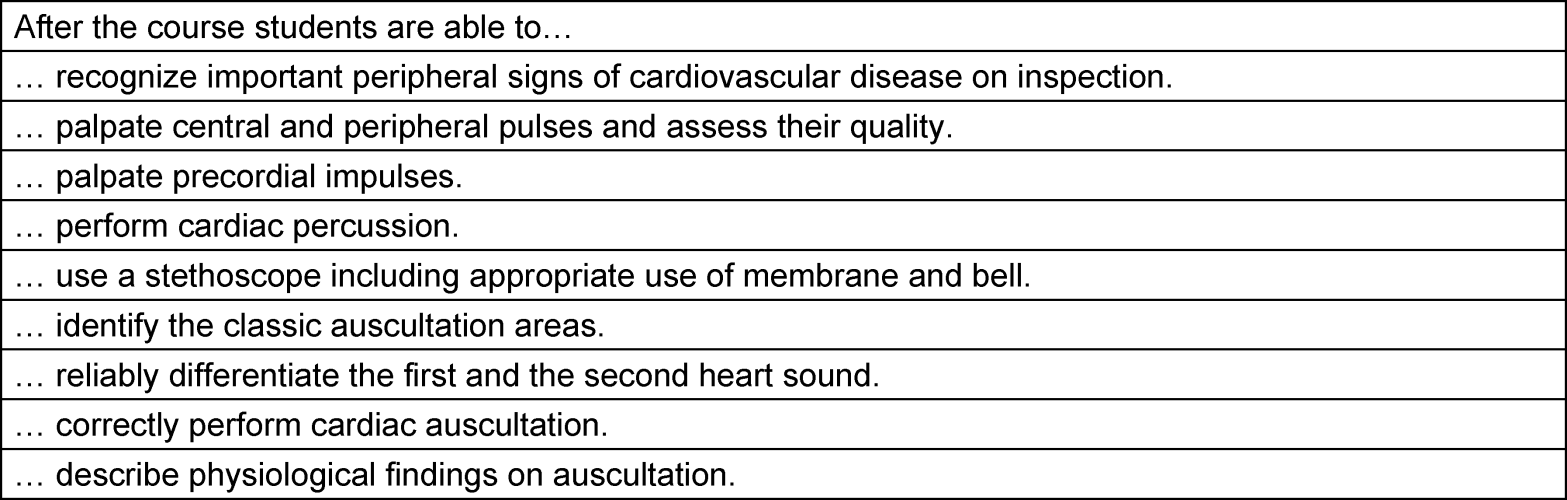
Learning objectives of the peer-led, simulator-based course

**Table 2:**
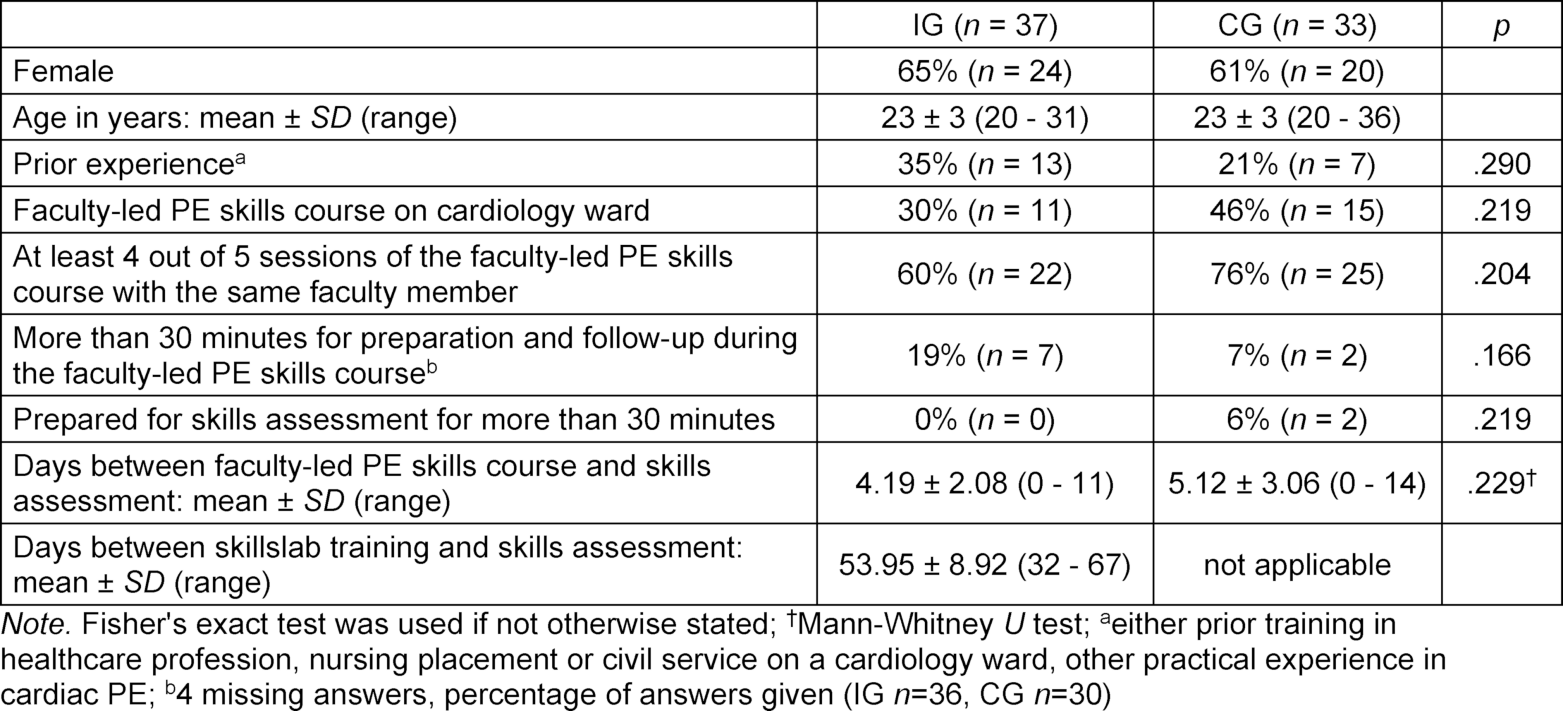
Descriptive statistics for intervention group (IG) and control group (CG)

**Table 3:**
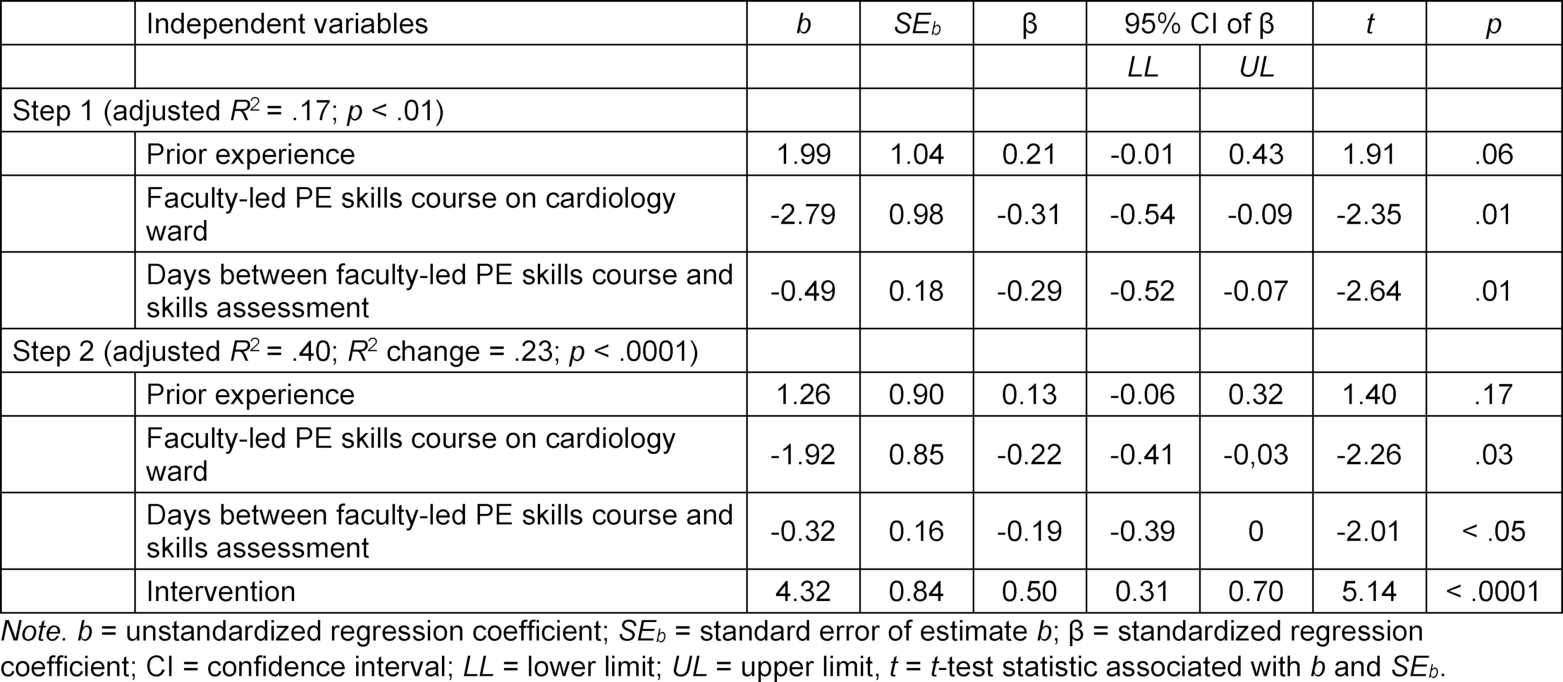
Relationship between possible confounders and performance in skills assessment

## References

[1] Zaman JA (2018). The Enduring Value of the Physical Examination. Med Clin North Am.

[2] Mangione S (2001). Cardiac auscultatory skills of physicians-in-training: a comparison of three English-speaking countries. Am J Med.

[3] Vukanovic-Criley JM, Criley S, Warde CM, Boker JR, Guevara-Matheus L, Churchill WH, Nelson WP, Criley JM (2006). Competency in cardiac examination skills in medical students, trainees, physicians, and faculty: a multicenter study. Arch Intern Med.

[4] Nielsen T, Mølgaard H, Ringsted C, Eika B (2010). The development of a new cardiac auscultation test: How do screening and diagnostic skills differ?. Med Teach.

[5] Lam MZ, Lee TJ, Boey PY, Ng WF, Hey HW, Ho KY, Cheong PY (2005). Factors influencing cardiac auscultation proficiency in physician trainees. Singapore Med J.

[6] Issenberg SB, McGaghie WC, Petrusa ER, Lee Gordon D, Scalese RJ (2005). Features and uses of high-fidelity medical simulations that lead to effective learning: a BEME systematic review. Med Teach.

[7] Cook DA, Hatala R, Brydges R, Zendejas B, Szostek JH, Wang AT, Erwin PJ, Hamstra SJ (2011). Technology-enhanced simulation for health professions education: a systematic review and meta-analysis. JAMA.

[8] Ewy GA, Felner JM, Juul D, Mayer JW, Sajid AW, Waugh RA (1987). Test of a cardiology patient simulator with students in fourth-year electives. J Med Educ.

[9] Gordon MS, Ewy GA, DeLeon AC, Waugh RA, Felner JM, Forker AD, Gessner ICH, Mayer JW, Patterson D (1980). "Harvey," the cardiology patient simulator: pilot studies on teaching effectiveness. Am J Cardiol.

[10] Kern DH, Mainous AG, Carey M, Beddingfield A (2011). Simulation-based teaching to improve cardiovascular exam skills performance among third-year medical students. Teach Learn Med.

[11] Butter J, McGaghie WC, Cohen ER, Kaye M, Wayne DB (2010). Simulation-based mastery learning improves cardiac auscultation skills in medical students. J Gen Intern Med.

[12] Fraser K, Wright B, Girard L, Tworek J, Paget M, Welikovich L, McLaughlin K (2011). Simulation training improves diagnostic performance on a real patient with similar clinical findings. Chest.

[13] Bernardi S, Giudici F, Leone MF, Zuolo G, Furlotti S, Carretta R, Fabris B (2019). A prospective study on the efficacy of patient simulation in heart and lung auscultation. BMC Med Educ.

[14] Parry J, Mathers J, Thomas H, Lilford R, Stevens A, Spurgeon P (2008). More students, less capacity? An assessment of the competing demands on academic medical staff. Med Educ.

[15] Topping KJ (1996). The effectiveness of peer tutoring in further and higher education: A typology and review of the literature. High Educ.

[16] Hughes TC, Jiwaji Z, Lally K, Lloyd-Lavery A, Lota A, Dale A, Janas R, bulstrode CJ (2010). Advanced Cardiac Resuscitation Evaluation (ACRE): a randomised single-blind controlled trial of peer-led vs. expert-led advanced resuscitation training. Scand J Trauma Resusc Emerg Med.

[17] Graham K, Burke JM, Field M (2008). Undergraduate rheumatology: can peer-assisted learning by medical students deliver equivalent training to that provided by specialist staff?. Rheumatology (Oxford).

[18] Weyrich P, Celebi N, Schrauth M, Möltner A, Lammerding-Köppel M, Nikendei C (2009). Peer-assisted versus faculty staff-led skills laboratory training: a randomised controlled trial. Med Educ.

[19] Haist SA, Wilson JF, Fosson SE, Brigham NL (1997). Are fourth-year medical students effective teachers of the physical examination to first-year medical students?. J Gen Intern Med.

[20] Herrmann-Werner A, Gramer R, Erschens R, Nikendei C, Wosnik A, Griewatz J, Zipfel S, Junne F (2017). Peer-assisted learning (PAL) in undergraduate medical education: An overview. Z Evid Fortbild Qual Gesundhwes.

[21] Takashina T, Shimizu M, Katayama H (1997). A new cardiology patient simulator. Cardiology.

[22] O'Rourke R, Braunwald E, Kasper D, Braunwald E, Hauser S, Longo D, Jameson J, Fauci A (2005). Physical examination of the cardiovascular system. Harrison's principles of internal medicine.

[23] Chizner MA (2001). The diagnosis of heart disease by clinical assessment alone. Curr Probl Cardiol.

[24] Hatala R, Scalese RJ, Cole G, Bacchus M, Kassen B, Issenberg SB (2009). Development and validation of a cardiac findings checklist for use with simulator-based assessments of cardiac physical examination competence. Simul Healthc.

[25] Kronschnabl DM, Baerwald C, Rotzoll DE (2021). Data from: Evaluating the effectiveness of a structured, simulator-assisted, peer-led training on cardiovascular physical examination in third-year medical students: a prospective, randomized, controlled trial.

[26] McKinney J, Cook DA, Wood D, Hatala R (2013). Simulation-based training for cardiac auscultation skills: systematic review and meta-analysis. J Gen Intern Med.

[27] Whipple ME, Barlow CB, Smith S, Goldstein EA (2006). Early introduction of clinical skills improves medical student comfort at the start of third-year clerkships. Acad Med.

[28] Hatala R, Issenberg SB, Kassen BO, Cole G, Bacchus CM, Scalese RJ (2007). Assessing the relationship between cardiac physical examination technique and accurate bedside diagnosis during an objective structured clinical examination (OSCE). Acad Med.

[29] Krautter M, Diefenbacher K, Koehl-Hackert N, Buss B, Nagelmann L, Herzog W, Jünger J, Nikendei C (2015). Short communication: final year students' deficits in physical examination skills performance in Germany. Z Evid Fortbild Qual Gesundhwes.

[30] Störmann S, Stankiewicz M, Raes P, Berchtold C, Kosanke Y, Illes G, Loose P, Angstwurm MW (2016). How well do final year undergraduate medical students master practical clinical skills?. GMS J Med Educ.

[31] Haring CM, Cools BM, van der Meer JW, Postma CT (2014). Student performance of the general physical examination in internal medicine: an observational study. BMC Med Educ.

[32] Verghese A, Charlton B, Kassirer JP, Ramsey M, Ioannidis JP (2015). Inadequacies of Physical Examination as a Cause of Medical Errors and Adverse Events: A Collection of Vignettes. Am J Med.

[33] Field M, Burke JM, McAllister D, Lloyd DM (2007). Peer-assisted learning: a novel approach to clinical skills learning for medical students. Med Educ.

[34] Weyrich P, Schrauth M, Kraus B, Habermehl D, Netzhammer N, Zipfel S, Jünger J, Riessen R, Nikendei C (2008). Undergraduate technical skills training guided by student tutors--analysis of tutors' attitudes, tutees' acceptance and learning progress in an innovative teaching model. BMC Med Educ.

[35] Chenot JF, Simmenroth-Nayda A, Koch A, Fischer T, Scherer M, Emmert B, Stanske B, Kochen MM, Himmel W (2007). Can student tutors act as examiners in an objective structured clinical examination?. Med Educ.

