# Supplementary material for: Evaluating the effectiveness of a structured, simulator-assisted, peer-led training on cardiovascular physical examination in third-year medical students: a prospective, randomized, controlled trial
Source: GMS J Med Educ. 2021 Sep 15;38(6):Doc108. doi: 10.3205/zma001504 (PMC8493837; doi:10.3205/zma001504)
Supplement: Student questionnaire [file JME-38-6-108-s-001.pdf]

Attachment 1: Student questionnaire

1. Personal Data
    1.1 What is your study ID-number?
    1.2 Please state your gender.
    1.3 What year were you born?
    1.4 What was the number of your study group during the faculty-led physical examination skills course?
2. Previous knowledge and experience
    2.1 Have you completed training in a healthcare profession in the past?
    2.2 If yes in 2.1, please state the profession.
    2.3 Have you had a nursing placement on a cardiology ward or were you on a cardiology ward as part of your civil service?
    2.4 Have you in the past gained any other practical experience in physical examination outside of your studies (e.g. during spare-time work)?
    2.5 If yes in 2.4, where?
3. Faculty-led internal medicine physical examination skills course
    3.1 On which ward was the faculty-led physical examination skills course conducted for your study group?
    3.2 How many of the five sessions where held by the same faculty member?
    3.3 How much time did you spend on preparation and follow up during the faculty-led physical examination skills course?
        - Up to 30 minutes
        - 30 to 59 minutes
        - 60 to 120 minutes
        - More than 120 minutes
4. Preparation for the skills assessment
    4.1 Have you prepared for this assessment? If yes, for how long did you prepare?
        - No
        - Up to 30 minutes
        - 30 to 59 minutes
        - 60 to 120 minutes
        - More than 120 minutes
    4.2 Which resources did you use to prepare?
        4.2.1 Textbook
        4.2.2 Internet research
        4.2.3 Lecture manuscripts
        4.2.4 Other

Attachment 1 to: Kronschnabl DM, Baerwald C, Rotzoll DE. *Evaluating the effectiveness of a structured, simulator-assisted, peer-led training on cardiovascular physical examination in third-year medical students: a prospective, randomized, controlled trial*. GMS J Med Educ. 2021;38(6):Doc108. DOI: 10.3205/001504
